# Alteration of glycine receptor immunoreactivity in the auditory brainstem of mice following three months of exposure to radiofrequency radiation at SAR 4.0 W/kg

**DOI:** 10.3892/ijmm.2014.1784

**Published:** 2014-05-22

**Authors:** DHIRAJ MASKEY, HYUNG GUN KIM, MYUNG-WHAN SUH, GU SEOB ROH, MYEUNG JU KIM

**Affiliations:** 1 Department of Anatomy, Dankook University College of Medicine, Cheonan-si, Chungnam, Republic of Korea; 2 Department of Pharmacology, Dankook University College of Medicine, Cheonan-si, Chungnam, Republic of Korea; 3 Department of Otorhinolaryngology, Seoul National University Hospital, Jongno-gu, Seoul, Republic of Korea; 4 Department of Anatomy, Institute of Health Sciences, Gyeongsang National University School of Medicine, Jinju, Gyeongsang, Republic of Korea

**Keywords:** radiofrequency, glycine receptor, superior olivary complex, cochlear nuclear complex, nucleus of lateral lemniscus, inferior colliculus

## Abstract

The increasing use of mobile communication has triggered an interest in its possible effects on the regulation of neurotransmitter signals. Due to the close proximity of mobile phones to hearing-related brain regions during usage, its use may lead to a decrease in the ability to segregate sounds, leading to serious auditory dysfunction caused by the prolonged exposure to radiofrequency (RF) radiation. The interplay among auditory processing, excitation and inhibitory molecule interactions plays a major role in auditory function. In particular, inhibitory molecules, such a glycine, are predominantly localized in the auditory brainstem. However, the effects of exposure to RF radiation on auditory function have not been reported to date. Thus, the aim of the present study was to investigate the effects of exposure to RF radiation on glycine receptor (GlyR) immunoreactivity (IR) in the auditory brainstem region at 835 MHz with a specific absorption rate of 4.0 W/kg for three months using free-floating immunohistochemistry. Compared with the sham control (SC) group, a significant loss of staining intensity of neuropils and cells in the different subdivisions of the auditory brainstem regions was observed in the mice exposed to RF radiation (E4 group). A decrease in the number of GlyR immunoreactive cells was also noted in the cochlear nuclear complex [anteroventral cochlear nucleus (AVCN), 31.09%; dorsal cochlear nucleus (DCN), 14.08%; posteroventral cochlear nucleus (PVCN), 32.79%] and the superior olivary complex (SOC) [lateral superior olivary nucleus (LSO), 36.85%; superior paraolivary nucleus (SPN), 24.33%, medial superior olivary nucleus (MSO), 23.23%; medial nucleus of the trapezoid body (MNTB), 10.15%] of the mice in the E4 group. Auditory brainstem response (ABR) analysis also revealed a significant threshold elevation of in the exposed (E4) group, which may be associated with auditory dysfunction. The present study suggests that the auditory brainstem region is susceptible to chronic exposure to RF radiation, which may affect the function of the central auditory system.

## Introduction

The unprecedented growth in the field of telecommunications has raised concerns about health risks associated with exposure to radiofrequency (RF) radiation. Frequent mobile users constantly complain of headaches, a burning sensation after an extended duration of communication (1), as well as sleep disturbances (2). RF radiation has been reported to have an impact on neuronal function, including the regulation of synaptic plasticity, neurotransmitter release, neuronal survival and learning and memory (3), since RF radiation possibly induces a decrease in the neuronal cell number and affects brain activity (3–5). Following exposure to RF radiation, neuronal loss and damage have been observed in the cerebellum, basal ganglia and hippocampus (4,5). A decrease in the number of of pyramidal cells in the cornu ammonis (CA) areas and the dentate gyrus has also been demonstrated after three months of exposure to a specific absorption rate (SAR) of 1.6 W/kg at an RF of 835 MHz (6) and 28 days (1 h/day) of whole body exposure to SAR 0.016 and exposure of the head to SAR 2 W/kg at 900 MHz electromagnetic field (EMF) (7), providing clues as to the considerable effects of exposure to RF radiation on living organisms. In addition to neuronal cells, glial cells, particularly astrocytes, are also activated by exposure to RF radiation in the cortex, caudate putamen, striatum, hippocampus and the cerebellum (8,9). A study employing low-level frequency exposure for 90 days to 50 Hz revealed alterations in the activity of the central nervous system (CNS), affecting the regulation of Ca^2+^ and N-methyl-D-aspartate (NMDA) receptor activity, suggesting perturbed neuronal functions due to exposure to EMF (3). Exposure to pulsed RF radiation (900 MHz at SAR 4.0 W/kg) has shown a selective diminution of rat Purkinje cell processes immunostained by γ-aminobutyric acid (GABA)-specific antibody (10). GABA immunoreactivity (IR) in the three layers of the rat cerebellar cortex was also decreased by continuous exposure to RF radiation (900 MHz at 32 W/kg) (10).

The interplay between neuronal function, excitation and inhibitory inputs at the central level plays a major role in auditory processing (11) since the excitatory responses induced by sound stimulation on one side are counteracted by the inhibition obtained from stimulation on the other side (12). Inhibitory interactions based on neuronal networks crucially depend on the GABA or glycine, acting as transmitters within the auditory system (11). Glycine, similar to GABA, is a major inhibitory neurotransmitter, predominantly localized in the brainstem (13). Glycinergic inputs are involved in sound localization or lateral inhibition (11) and play a major role in affecting the development of post-synaptic properties (14), as well as in the refinement of synaptic connections during the postnatal period (15). In addition, glycinergic inputs are essential to auditory function in the auditory nuclei of the brainstem (16) from the cochlear nuclear complex (CNC) to the inferior colliculus (IC) (17,18).

Auditory information of the ascending and descending signal is processed by various nuclei situated along the auditory brainstem including, the CNC, the superior olivary complex (SOC), nuclei of the lateral lemniscus (NLL) and the IC, each possessing distinct properties as regards activation (19). The CNC is composed of the dorsal cochlear nucleus (DCN) and the ventral cochlear nucleus with further divisions of the ventral nuclei into anteroventral cochlear nuclei (AVCN) and posteroventral cochlear nuclei (PVCN) (20). Fibers from the cells of the cochlear nuclei comprise parallel ascending pathways to the SOC, while it receives morphologically distinct axosomatic endings of primary afferent fibers from the cochlea (21).

The SOC as a part of the auditory system is comprised of the lateral superior olive (LSO), the superior paraolivary nucleus (SPN) and the medial nucleus of the trapezoid body (MNTB) (22). The LSO receives bilateral innervation and comprises an important part of hearing as it is at this level in the ascending auditory pathway where binaural processing of sound localization cues first occurs (23). The MNTB provides inhibitory input to the LSO and imparts sensitivity to interaural intensity differences in cells in the LSO (24).

The IC is an obligatory processing station in the auditory pathway receiving inhibitory and excitatory afferents from the majority of the brainstem nuclei (25,26). The ascending auditory fibers end in an orderly tonotopic array of fibrodendritic layers of the central nucleus of the IC (ICC) (27).

Glycinergic synapse is known to occur throughout the auditory brainstem, exerting inhibitory control over the discharge of auditory neurons through the action of the post-synaptic glycine receptors (GlyRs) (13,28,29). Changes in receptor expression may have an impact on synaptic strength (30). The nuclei of the SOC, mainly LSO, medial superior olivary nucleus (MSO) and SPN along with cochlear nuclei, provide the glycinergic input, while in the IC, these glycinergic inputs are provided by projections from the ventral nuclei of the lateral lemniscus (VNLL) and LSO. In addition, the glycinergic input is strongly provided to the SPN by the MNTB (31), whose neurons display offset responses to pure tones. The release from MNTB-derived glycinergic inhibition is critical to the formation of SPN offset responses (32). Hence, any disturbance in the auditory circuit region is likely to affect the normal hearing process. Previous reports have shown that a decrease in the number of GlyR immunoreactive cells in the auditory system is associated with hearing loss (22,33).

Considering that the use of the cellular phone entails its close proximity with hearing regions, exposure to RF radiation may induce changes in the neurotransmitters of hearing-related brain regions, including the central auditory nucleus. However, few studies have focused on the auditory system; furthermore, the majority of studies have concentrated on otoacoustic emission in both humans (34) and animals (35,36). Moreover, to the best of our knowledge, studies employing immunohistochemical methods as a quantitative approach for the acquisition of crucial information as to the effects of exposure to RF radiation in the auditory brainstem region have not been conducted to date. Therefore, the present study focused on alterations in GlyR IR in the central auditory brainstem following exposure to RF radiation using free-floating immunohistochemistry to assess the possible effects of exposure to RF radiation on GlyR IR in the auditory region (IC, NLL, SOC and CNC) of mice following exposure to RF radiation for three months at SAR 4.0 W/kg.

## Materials and methods

### Animal experimentats

Six-week-old ICR male mice (n=20; weighing, 20–30 g) obtained from Orientbio, Inc., Sungnam-si, Korea) were obtained and kept in an animal room under controlled conditions (mean temperature, 22.5±1°C; humidity, 55±10%; 12-h light/dark cycle). Food (Samtako Bio Korea, Osan, Korea) and water were supplied *ad libitum*. The experimental procedures were reviewed and approved by the Dankook University Institutional Animal Care and Use Committee (DUIAC), which adheres to the guidelines issued by the Institution of Laboratory and Animal Resources (ILAR) and were performed in compliance with NIH guidelines for animal research.

### Exposure system

The exposure system (Wave Exposer V20) was used in this study (6). The Wave Exposer V20, which emits 835 MHz (equivalent to the Korean CDMA mobile phone frequency), was designed by the Division of Information Technology Engineering, Soonchunhyang University (Asan, Korea) (6). The SAR was adjusted to 1.6 and 4.0 W/kg, which is the same value as the electric field intensity between 59.56 and 94.18 V/m for muscle (0.92, 57 and 1020 kg/m^3^) on the 835 MHz CDMA frequency. Waves were generated and amplified in an electronic unit, and were eventually radiated by a pyramidal rectangular horn antenna connected by a waveguide to the coaxial transition. A standard mouse cage of 22 inches was used for the apparatus. The output powers of the horn antenna from the exposure apparatus were 2.5 W for SAR 1.6 W/kg and 6.3 W for SAR 4.0 W/kg. Electric field intensities due to SAR values were be calculated, and the power value was obtained by a computer simulation with a high frequency structure simulator (HFSS) manufactured by Ansoft, Co. (Pittsburgh, PA, USA). Five three-dimensional cylindrical MEMS antennas were used for the simulation. The simulation variable included both the location of the mouse and the distance from the horn aperture for freely-moving mice. The power was obtained by averaging the simulated peak electric field intensities from each mouse body. The wave exposure from the horn antenna to the mouse cage was provided by the wave absorption material (TDK ceramic absorber) which mimics the radiation exposure in an open environment and limits the influence the number of mice may have on exposure. To eliminate potential stress during exposure, the exposure apparatus used in the present study had a cooling system that did not exceed a temperature of 26°C during the exposure period as reported in our previous study (6). Likewise, the internal temperature, which is always denoted by the digital number in front of the instrument, was checked on a regular basis in order to maintain the optimum temperature at 24°C. Additionally, the exposure apparatus provides an automatic light system with a water feeder and no restriction in movement. To elucidate ambient noise levels in the present study, a sound-level meter NA-24 (Rion, Co., Ltd., Tokyo, Japan) was used to determine ambient noise levels both within the exposure apparatus and in the animal room during RF radiation. When steady-state noise levels were recorded on >5 consecutive trials, each noise level of the animal room and exposure apparatus during RF radiation was confirmed to range from 42.0 to 44.6 dB and from 56.4 to 58.2 dB, respectively.

### Experimental design

The mice were exposed to 835 MHz of radiation with an average SAR of 4.0 W/kg using the Wave Exposer V20. The mice were divided randomly into two groups (n=10): i) a sham control (SC) group and ii) a group exposed to SAR of 4.0 W/kg for three months [exposed (E4) group). The exposure duration was 8 h/day. Both the SC and E4 groups were subjected to the same surgical and anesthetic procedure for auditory brainstem response (ABR) testing.

### ABR

The ABR was recorded using a signal-processing system (System III, Tucker Davis Technologies, Alachua, FL, USA). The ABR recording was performed immediately after the three-month period of exposure to RF radiation. For anesthesia, zolazepam (Zoletil; Virbac, Carros Cedex, France) and xylazine (Rompun; Bayer, Leverkusen, Germany) were mixed in a 4:1 ratio (0.1 ml/100 g). During ABR recording, the animals were placed on a warm pad, the temperature of which was approximately 40°C. Acquired auditory-evoked brainstem responses were filtered through a 10-kHz high pass and a 3-kHz low pass filter. The sampling rate was 25 k samples/sec. The presented stimulus was a rarefaction click of 0.1 msec duration.

The animals were placed in a soundproof booth and three electrodes were inserted subcutaneously, one at the vertex and the other two ventrolaterally to each ear, beneath the pinna (active, reference and ground electrodes, respectively). The click stimuli were delivered through a tube inserted into the ear canal of the mouse. Hearing thresholds were determined by the assessment of the lowest stimulus level required to elicit the ABR peaks III or V at levels from 10 to 80 dB sound pressure level (SPL) in 5-dB steps. When the ABR threshold was >80 dB SPL, it was defined as 80 for statistical analysis. One thousand and twenty-four tone presentations were averaged. The ABR hearing thresholds were confirmed by an independent observer. Statistical procedures were implemented using Statistical Package for Social Sciences (SPSS) version 17 software. Statistical differences in the ABR thresholds between the SC and E4 groups were compared using the Mann-Whitney test. The difference between the groups was considered statistically significant at a value of P<0.05.

### Immunohistochemical analysis

The animals were anesthetized with diethyl ether and their brains were collected following transcardial perfusion with phosphate-buffered saline (PBS) and a 4% paraformaldehyde (PFA) solution. Anesthesia was also used to avoid animal stress and to lower the augmentation of blood pressure during perfusion and fixation. Following perfusion, the brains were immediately removed, post-fixed overnight in 4% PFA and cryoprotected by infiltration with a sucrose series (10, 20 and 30%) solution at 4°C. Serial coronal sections of 40 μm thickness were obtained using a cryocut microtome (CM3050S, Leica Biosystems Nussloch GmbH) and collected in 6-well plates. Immunohistochemistry was performed using a free-floating method, as previously described (6). Briefly, the coronal sections were incubated for 48 h at 4°C in rabbit polyclonal antibodies to GlyR α_1_+α_2_ (ab23809, dilution ratio of 1:2,500; Abcam, Cambridge, UK) in blocking buffer containing 1% bovine serum albumin, 0.3% Triton X-100, and 1% normal goat serum. To eliminate peroxidase activity, the sections were treated with 1% hydrogen peroxide in PBS. The sections were incubated with a biotinylated secondary antibody at the dilution ratio of 1:250 for 1.5 h at room temperature, followed by treatment with an avidin-biotin-peroxidase complex (Vectastain ABC mouse Elite kit; Vector Laboratories, Burlingame, CA, USA). Following three washes in PBS, the sections were reacted with 3,3′-diaminobenzidine (DAB) and hydrogen peroxide in a distilled water solution for 5 min. The sections from each group were stained together to minimize variability. A sample of sections was reacted without primary antiserum. The sections from these samples did not exhibit any of the IR described in this study. Following additional washes, dehydration in solutions of increasing percentages of ethanol and clearing in xylene, the sections were mounted on gelatin-coated slides with a cover slide for analysis.

### Image analysis

An Olympus BX51 microscope was used for analysis and images were acquired using a digital camera system (DP50; Olympus, Tokyo, Japan). The NIH image program (ImageJ, version 1.44) was used to determine the staining densities and was also used for cell counting using the manual cell counting and marking method. Only sections with clearly differentiated layers of nuclei from the brainstem auditory circuit were collected for densitometry analysis. Immunolabeling in the SC and E4 groups was carried out as previously described (6). Gray values of the digitized micrographs were analyzed within the outlines of all the identifiable nuclei within a section. The sum of the gray values of all pixels in each corresponding region was divided by the total number of pixels in the region to determine the mean density of IR per unit area (mm^2^). Only the clearly stained nuclei were included in the analysis. Outlined regions of interest for corresponding nuclei were analyzed with similar size while the out-of-focus structures and non-uniform stained figures were excluded (total of 50–100 nuclei per antibody and animal group). The background of each section was measured by outlining an area in the nearby tissue in which the stain was very weak. The analysis of the slides was performed by an investigator blinded to the experimental procedures.

### Statistical analysis

Differences between different nuclei from the auditory brainstem regions were determined by one-way analysis of variance (ANOVA) followed by post-hoc analysis with the Bonferroni test (SigmaPlot version 10.0; Systat Software Inc., Chicago, IL, USA). Student’s t-tests using SPSS software version 17 were used when only two groups were compared. Values are expressed as the means ± standard deviation (SD). A P-value <0.05 was considered to indicate a statistically significant difference.

## Results

### ABR

**Figure 1:**
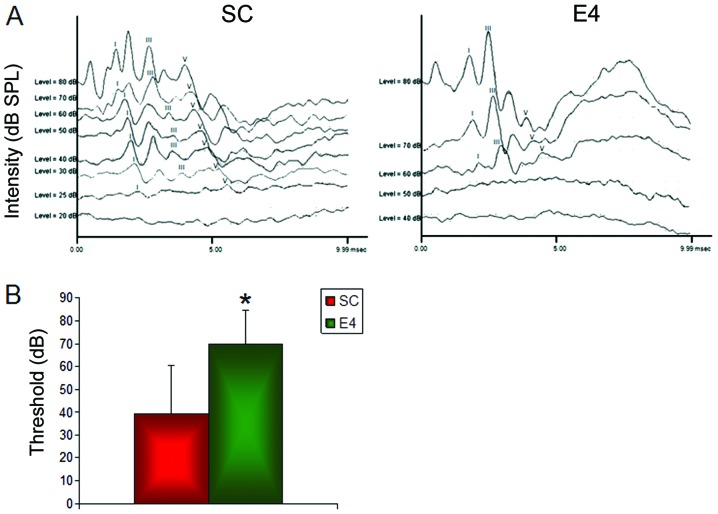
(A) Effect of radiofrequency (RF) exposure on the auditory brainstem response (ABR) thresholds on sham control (SC) group and the group exposed to RF radiation (E4 group) at SARs of 0 and 4.0 W/kg, respectively, after three months of exposure to RF radiation at 835 MHz. The SC group exhibited a characteristic ABR waveform at sound pressure levels of 25 dBm while the E4 group exhibited elevated levels of approximately 60 dB. (B) Mean ABR thresholds for the SC and E4 groups at SARs 0 and 4.0 W/kg, respectively, following three months of exposure to RF radiation at 835 MHz showed significant threshold elevations in the E4 group. The data shown are the means ± SD (^*^P<0.001).

The ABR response was detectable even with a very small stimulus intensity in the SC group, but was only detectable with a large stimulus intensity in the E4 group (Fig. 1A). The SC group exhibited characteristic ABR waveforms at an SPL as low as 25 dB, while the E4 group ABR threshold was measured at 60 dB. The ABR threshold of the SC group was 39.3±21.1 dB SPL, and that of the E4 group was 70.0±14.7 dB SPL (Fig. 1B). The ABR threshold of the E4 group was significantly higher than that of the SC group (P<0.001), signifying a decrease in hearing intensity in the E4 group (Fig. 1B).

### Histological observations

#### i) CNC

**Figure 2:**
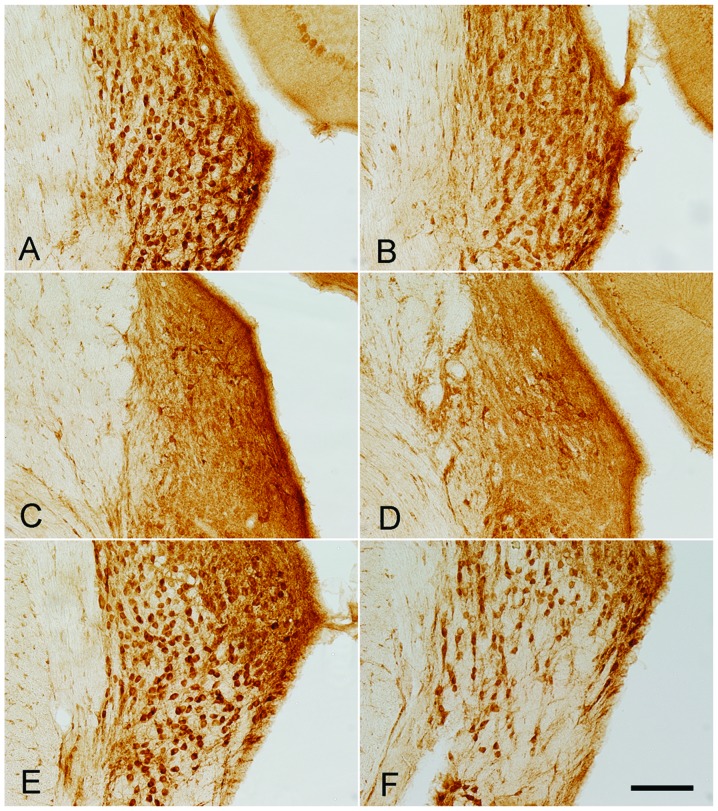
Photomicrograph of glycine receptor (GlyR) α_1_+α_2_ immunoreactivity (IR) in coronal sections of the cochlear nuclear complex comprising of (A and B) AVCN, (C and D) DCN and (E and F) PVCN of the (A, C and E) sham control (SC) group and (B, D and F) group exposed to radiofrequency (RF) radiation (E4 group) at SARs 0 and 4.0 W/kg, respectively, after three months of exposure to RF radiation at 835 MHz. A prominent decrease in GlyR IR of neuropils, as well as in the number of GlyR immunoreactive neurons was observed in the E4 group compared with the SC group. AVCN, anteroventral cochlear nucleus; PVCN, posteroventral cochlear nucleus; DCN, dorsal cochlear nucleus. Scale bar, 100 μm.

**Figure 3:**
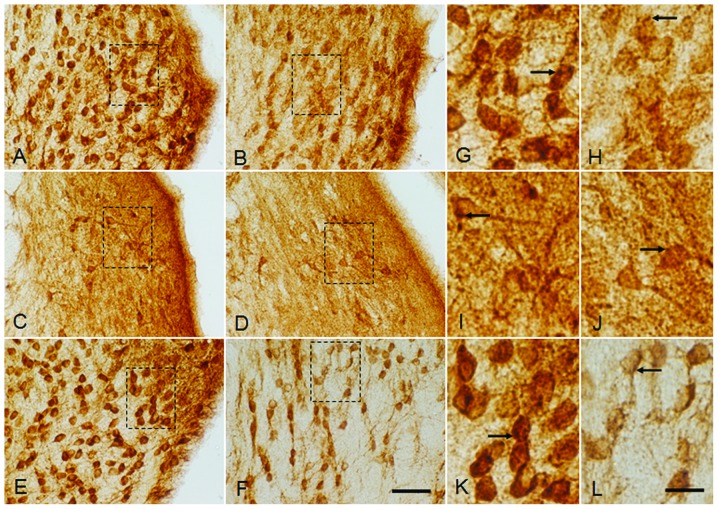
Magnified image of glycine receptor (GlyR) α_1_+α_2_ immunoreactivity (IR) in coronal sections through the cochlear nuclear complex, including the (A, B, G and H) AVCN, (G, D, I and J) DCN and (E, F, K and L) PVCN of the (A, C, E, G, I and K) sham control (SC) group and (B, D, F, H, J and L) the group exposed to radiofrequency radiation (E4 group) at SARs of 0 and 4.0 W/kg, respectively, after three months of exposure to RF radiation at 835 MHz. Magnified images of the dotted squares in (A, B, C, D, E and F) are represented in (G, H, I, J, K and L, respectively). Note the loss of GlyR IR in the somas of the E4 group in all the three regions of the cochlear nuclear complex, which was found to be very severe in the PVCN. The number of GlyR immunoreactive puncta (arrows) was also prominently reduced in the E4 group compared with the SC group. AVCN and PVCN showed bushy cells (BCs) (thick arrows) with lighter staining in the E4 group compared with the SC group. A variety of cell types present in the AVCN, DCN and PVCN showed decreased staining intensity in the E4 group as compared with SC group. AVCN, anteroventral cochlear nucleus; PVCN, posteroventral cochlear nucleus; DCN, dorsal cochlear nucleus. Scale bar: A–F, 50 μm; G–L, 10 μm.

GlyR IR was observed in the cells and neuropils in all the nuclei of the CNC (Fig. 2A–F). In the AVCN of the SC group, the GlyR IR was noted to be localized in the cell bodies and the dendrites of the bushy cells (BC) along with prominent GlyR immunoreactive puncta close to the cell membrane (Fig. 3A and G). Compared with the SC group, the GlyR IR of BCs was markedly decreased in th E4 group, particularly in the puncta, and was almost absent in some cells (Fig. 3B and H). The DCN, irrespective of its subdivisions, revealed prominent GlyR IR along with the primary dendrites and neuropils in the SC group and darkly stained puncta were close to the cell membrane (Fig. 3C and I). However, in the E4 group, the overall decrease in the GlyR immunoreactive cell number was accompanied by a reduction in cell size and a marked decrease in GlyR IR was also observed in the DCN. In addition, GlyR IR of puncta was also much decreased in the cells as well too (Fig. 3D and J). GlyR IR was identified in the fusiform (Fig. 3I and J) and cartwheel cells (Fig. 3I and J) of the DCN and GlyR IR of those cells was decreased in the E4 group compared with the SC group. Similarly, the PVCN of the SC group stained with the GlyR-specific antibody revealed prominent GlyR IR in the neuronal somas and puncta closely lining the cells. In the E4 group, compared to the SC group, GlyR IR of BCs in the PVCN was markedly decreased. The decrease in cell number in the E4 group compared with the SC group was clearly distinguishable and few GlyR immunorective cells in the PVCN of the E4 group clearly showed a decrease in GlyR IR and a decrease in staining intensity within the puncta (Fig. 3E, F, K and L). A decrease in the staining intensity of the neuropils in the E4 group (Fig. 3H, J and L) compared with the SC group (Fig. 3G, I and K) was also observed in all three subdivisions of the CNC.

According to the relative density, GlyR IR was significantly (P<0.05) decreased by 4.5% in the AVCN from 109.42±5.27 in the SC group to 104.41±5.82 in the E4 group (Fig. 9A). GlyR IR in the DCN region was 121.35±2.90 in the SC group which was significantly decreased (P<0.0001) by 5.68% to 114.45±3.24 in the E4 group (Fig. 9A). A significant (P<0.0001) decrease in GlyR IR in the PVCN was also observed from 103.96±4.62 in the SC group to 91.07±3.10 in the E4 group which amounted to 12.40% (Fig. 9A).

#### ii) SOC

**Figure 4:**
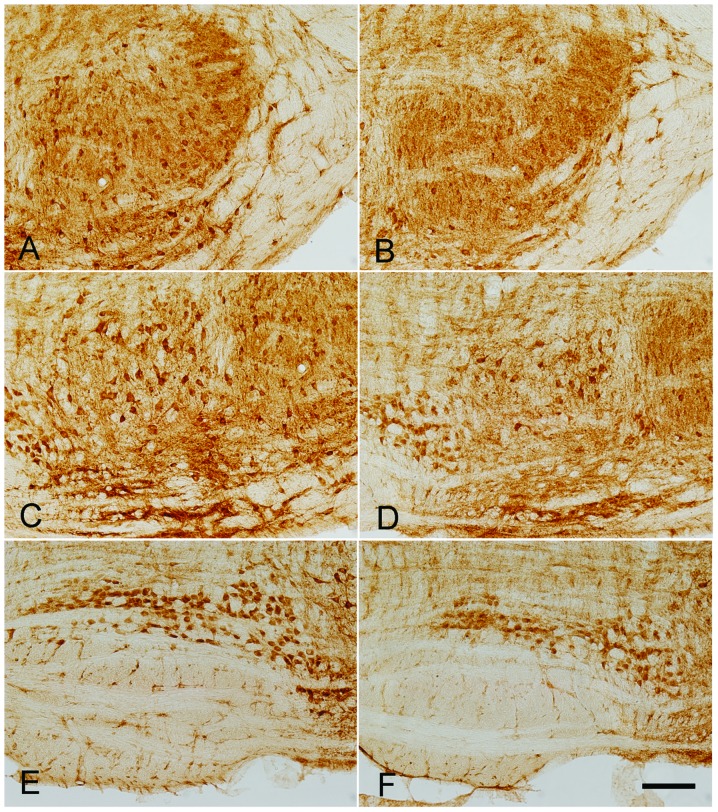
Photomicrograph of glycine receptor (GlyR) α_1_+α_2_ immunoreactivity (IR) in coronal sections of the SOC comprising of (A and B) LSO; (C and D) SPN and MSO; and (E and F) MNTB of (A, C and E) the sham control (SC) group and (B, D and F) group exposed to radioafrequency (RF) radiation (E4 group) at SARs 0 and 4.0 W/kg, respectively, after three months of exposure to RF radiation at 835 MHz. GlyR IR was observed in the neurons and neuropils of the (A and B) LSO; (C and D) SPN and MSO; and (E and F) the MNTB of both the SC and E4 groups. Comparisons with the SC group revealed a loss inf GlyR IR in all the nuclei as well as the neuropils of the E4 group. SOC, superior olivary complex; LSO, lateral superior olive; SPN, superior paraolivary nucleus; MSO, medial superior olive; MNTB, medial nucleus of the trapezoid body. Scale bar, 100 μm.

**Figure 5:**
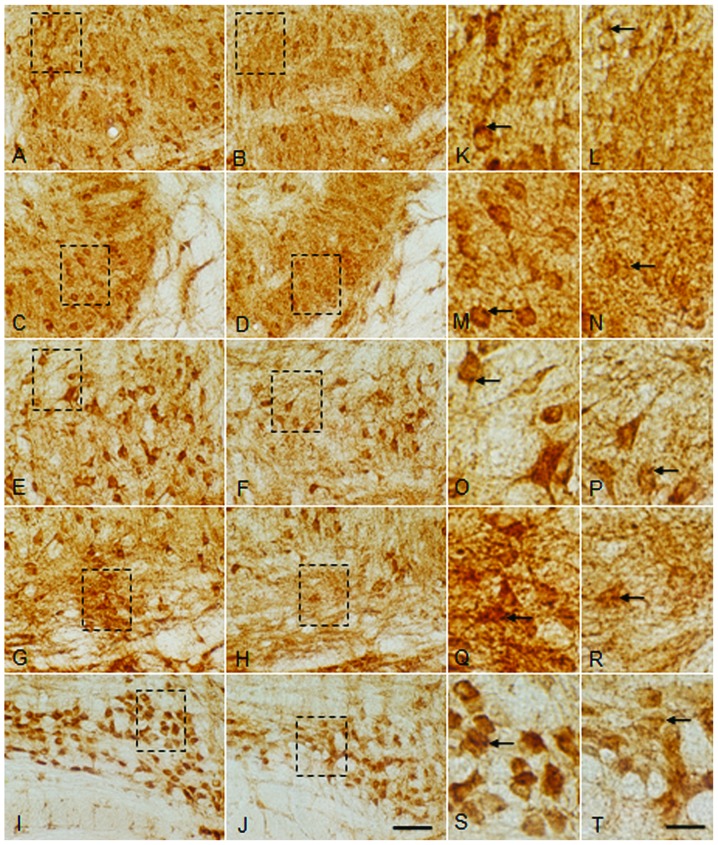
Magnified image of glycine receptor (GlyR) α_1_+α_2_ immunoreactivity (IR) in coronal sections through the SOC composed of the (A–D and K–L) LSO, (E, F, O, P and L) SPN, (G, H, Q and R) MSO and (G, H, S and T) MNTB of (A, C, E, G, H, K, M, O, Q and S) the sham control (SC) group and (B, D, F, H, J, L, N, P, R and T) the group exposed to radiofrequency radiation (E4 group) at SARs 0 and 4.0 W/kg, respectively, after three months of exposure RF radiation at 835 MHz. Magnified images of the dotted squares in (A, B, C, D, E, F, G, H, I and J) are represented in (K, L, M, N, O, P, Q, R, S and T, respectively). Scattered highly GlyR immunoreactive cells were noted in all the nuclei of the SOC. Puncta (arrows) representing immunoreactive presynaptic terminals were also noted. Loss of the GlyR immunoreactive cells was noted in the (B and L) medial and (D and N) lateral area of the LSO, (F and P) SPN, (H and R) MSO and (J and T) MNTB of the E4 group compared with the SC group. Also note the decrease in staining intensity of the puncta (arrows) of the E4 group compared with the SC group. SOC, superior olivary complex; LSO, lateral superior olive; SPN, superior paraolivary nucleus; MSO, medial superior olive; MNTB, medial nucleus of the trapezoid body. Scale bar: A–H, 50 μm; I–P, 10 μm.

GlyR IR was noted in all the major nuclei of the SOC, most notably in the LSO, SPN, MSO and MNTB of the SC group. GlyR IR was localized in both the somas and neuropils of the LSO, SPN and MSO, while the somas were stained mainly in the MNTB region (Fig. 4). Prominent GlyR immunoreactive puncta were observed as close to the membrane of the soma leaving the perikarya unstained. The GlyR immunoreactive puncta were restricted to the soma and were not detected in the dendrites or neuropils (Fig. 5K–T). In staining with GlyR-specific antibody, the bipolar cells with the eccentric nucleus were mainly found in the LSO and MSO, while the SPN comprised of GlyR immunoreactive bipolar, as well as several multipolar neurons. The SPN and MSO contained numerous GlyR immunoreactive fibers in between the GlyR immunoreactive cells (Fig. 5O–R). GlyR IR in the MNTB was specifically localized on the cellular membrane with GlyR immunoreactive puncta. The somas on the lateral aspect of the MNTB were prominently visible by staining with GlyR-specific antibody and staining was observed in the cytoplasm of the soma.

As compared to the SC group, a marked decrease in GlyR IR in the somas and neuropils was observed in the E4 group. In the LSO of the E4 group, particularly in the lateral limb, a decrease in GlyR IR in the cells of the E4 group and a loss of neuropil staining was observed (Fig. 5K–N). Similarly, a marked decrease in GlyR IR was observed in the SPN, MSO and MNTB of the E4 group (Fig. 5E–J). A significant decrease in the size of the soma with a loss of GlyR immunoreactive puncta was observed in the SPN of the E4 group (Fig. 5O and P). Furthermore, as compared with the SC group, the loss of GlyR IR in the soma was observed in the MSO of the E4 group (Fig. 5Q and R) and a marked decrease in GlyR IR in the fibers of the SPN, MSO and MNTB was also observed in the E4 group (Fig. 5I, J, S and T).

The relative mean density analysis revealed that GlyR IR in the LSO was 116.34±4.45 in the SC group, which was significantly decreased (P<0.0001) by 6.67% (108.57±3.39) in the E4 group (Fig. 9B). Compared with the SC group, measured as 99.22±2.94, a slight non-significant decrease to 98.37±2.08 in GlyR IR was observed in the E4 group, which amounted to a 0.85% decrease (Fig. 9B). A significant decrease (P<0.0001) in 10.49% in the SC group from 128.15±3.65 to 114.70±2.66 in the E4 group was noted in the MSO. The MNTB also exhibited a significant decrease in GlyR IR (P<0.05) of 6.23% from 100.87±3.86 in the SC group to 94.59±7.13 in the E4 group (Fig. 9B).

#### iii) NLL

**Figure 6:**
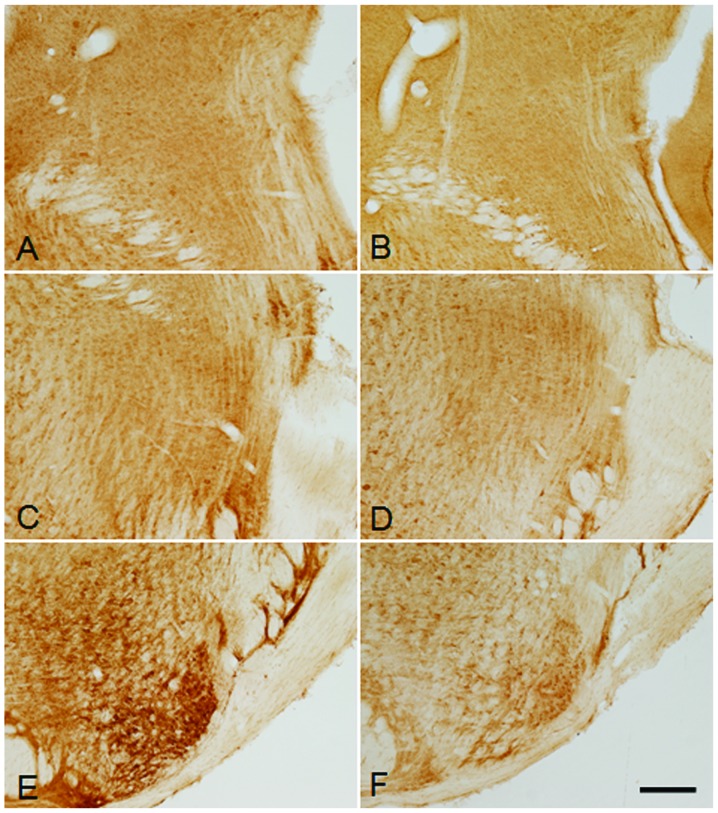
Photomicrograph of the glycine receptor (GlyR) α_1_+α_2_ immunoreactivity (IR) in coronal sections of the (A and B) DNLL, (C and D) INLL and (E and F) VNLL of the (A, C and E) sham control (SC) group and (B, D and F) the group exposed to radiofrequency (RF) radiation (E4 group) at SARs 0 and 4.0 W/kg, respectively after three months of exposure to RF radiation at 835 MHz. GlyR IR was noted in all three subdivisions of LL in the neuropils and neurons with varying intensity between the SC and E4 groups. The reduction in GlyR staining intensity was noted to be very severe in the VNLL. DNLL, dorsal nucleus of lateral leminiscus; INLL, intermediate nucleus of lateral leminiscus; VNLL, ventral nucleus of lateral leminiscus; NLL, nucleus of lateral leminiscus. Scale bar: A–F, 100 μm.

**Figure 7:**
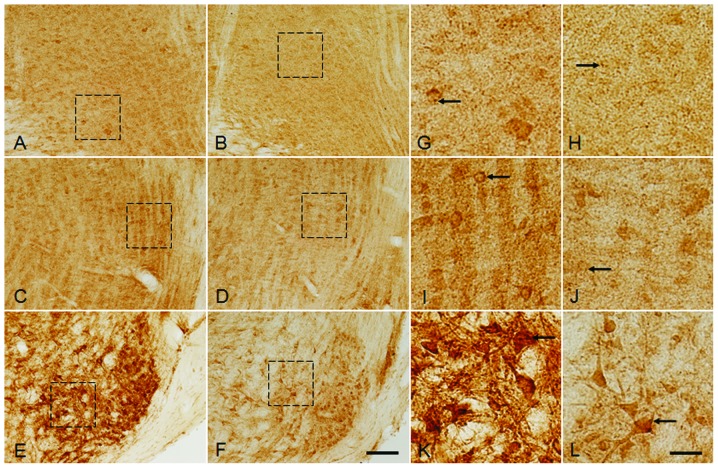
Magnified image of glycine receptor (GlyR) α_1_+α_2_ immunoreactivity (IR) in coronal sections through the (A, B, G and H) DNLL, (C, D, I and J) INLL and (E, F, K and L) VNLL of the (A, C, E, G, I and K) sham control (SC) group and (B, D, F, H, J and L) the group exposed to radiofrequency radiation (E4 group) at SARs 0 and 4.0 W/kg, respectively, after three months of exposure to RF radiation at 835 MHz. Magnified images of the dotted squares in (A, B, C, D, E and F) are represented in (G, H, I, J, K and L, respectively). The GlyR immunoreactive soma were weakly stained in the subdivisions of the NLL of the E4 group as compared with the SC group. Note the decrease in the staining intensity of GlyR immunoreactive puncta (arrows) in the E4 group as well. DNLL, dorsal nucleus of lateral leminiscus; INLL, intermediate nucleus of lateral leminiscus; VNLL, ventral nucleus of lateral leminiscus; NLL, nucleus of lateral leminiscus. Scale bar: A–F, 50 μm; G–L, 10 μm.

In the SC group (Fig. 6), varying intensities of GlyR IR were observed in all three subdivisions of the NLL [VNLL, intermediate NLL (INLL) and dorsal NLL (DNLL)] localized in the somas and neuropils. Compared with the DNLL and INLL in both groups (Fig. 7A–J), the number of GlyR immunoreative soma of the VNLL was increased (Fig. 7E, F, K and L). In the SC group, the VNLL exhibited a higher density of GlyR immunoreactive soma than the soma in the DNLL and INLL (Fig. 7A–F). GlyR immunoreactive neuropils were prominently observed in the VNLL (Fig. 7A–F). Prominent GlyR immunoreactive puncta were also observed in the soma of the VNLL (Fig. 7K), while the number of GlyR immunoreactive puncta in the DNLL and INLL was markedly decreased (Fig. 7G and I). In the E4 group, the staining intensity of the GlyR was markedly decreased in all subdivisions, notably in the VNLL of the E4 group (Fig. 7F and L), compared with the SC group.

The relative mean density analysis revealed the highest level of GlyR IR in the VNLL and the lowest level in the DNLL in both groups. GlyR IR was markedly decreased in the E4 group as compared with the SC group. GlyR IR in the DNLL was 101.72±1.86 in the SC group, but was significantly decreased (P<0.0001) by 9.38% (to 92.17±2.01) in the E4 group (Fig. 9C). A significant decrease (P<0.0001) of 6.97% from 106.17±2.26 in the SC group to 98.76±1.96 in the E4 group was noted in the INLL (Fig. 9C). The VNLL also exhibited a significant decrease in GlyR IR (P<0.0001) of 13.76% from 126.87±2.29 in the SC group to 109.41±3.33 in the E4 group (Fig. 9C).

#### iv) IC

**Figure 8:**
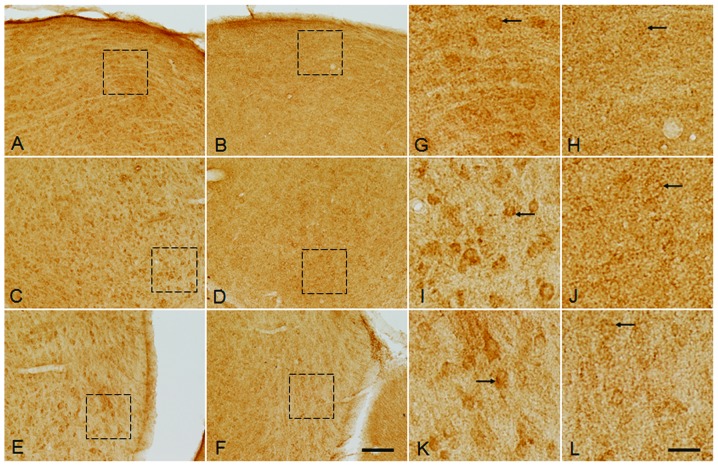
Photomicrograph of glycine receptor (GlyR) α_1_+α_2_ immunoreactivity (IR) in the coronal sections of the inferior colliculus (IC) composed of the (A, B, G and H) DC, (C, D, I and J) ICC and the (E, F, K and L) LN of the (A, C, E, G, I and K) sham control (SC) group and (B, D, F, H, J and L) the group exposed to radiofrequency radiation (E4 group) at SARs 0 and 4.0 W/kg, respectively, after three months of exposure to (RF) radiation at 835 MHz. Magnified images of the dotted squares in (A, B, C, D, E and F) are represented in (G, H, I, J, K and L, respectively). Note the prominent loss of GlyR IR in the immunoreactive soma (arrows) in all the regions of the IC of the E4 groups as compared with the SC group. DC, dorsal cortex of IC; ICC, central nucleus of IC; LN, lateral nucleus of IC. Scale bar: A–F, 100 μm; G–L, 10 μm.

In both the SC and E4 groups, the overall intensity of GlyR IR throughout the IC was observed to be lower than that in the SOC and CNC (Fig. 8A–F). In the SC group, all three subdivisions of the IC, the dorsal cortex (DC), ICC and lateral nucleus of the IC (LN) consisted of numerous GlyR immunoreactive neurons and dense GlyR immunoreactive puncta, which were mainly observed in the somas (Fig. 8A, C and E). In particular, GlyR immunoreactive bipolar neurons were observed in the SC group with specific dendritic distribution. When the E4 group was compared with the SC group, it was difficult to find the GlyR immunoreactive cells throughout the IC. Along with decreased number of GlyR immunoreactive neurons in all subdivisions of the IC, a marked decrease in GlyR immunoreactive soma was observed in the E4 group as well (Fig. 8G–L). In addition, GlyR IR intensity of the puncta was markedly decreased in the somas (Fig. 8G–L).

**Figure 9:**
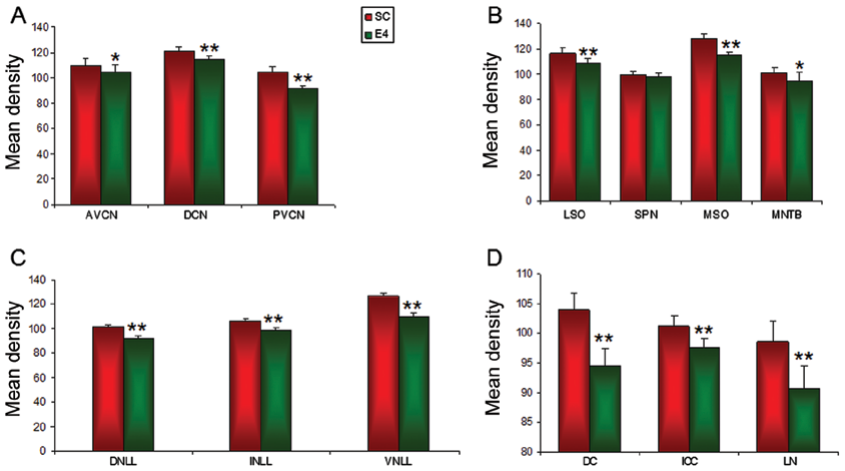
Image analysis of relative densities of glycine receptor (GlyR) α_1_+α_2_ immunoreactivity (IR) in the (A) IC, (B) NLL, (C) SOC and (D) CNC of the sham control (SC) group and the group exposed to radiofrequency radiation (E4 group) at SARs 0 and 4.0 W/kg, respectively after three months of exposure to RF radiation at 835 MHz. A decrease in IR was noted in the E4 group in the various regions of the auditory brainstem as compared with the SC group. The data shown are the means ± SD obtained from 5 different experiments. CNC, cochlear nuclear complex; AVCN, anteroventral cochlear nucleus; PVCN, posteroventral cochlear nucleus; DCN, dorsal cochlear nucleus; SOC, superior olivary complex; LSO, lateral superior olive; SPN, superior paraolivary nucleus; MSO, medial superior olive; MNTB, medial nucleus of the trapezoid body; DNLL, dorsal nucleus of lateral leminiscus; INLL, intermediate nucleus of lateral leminiscus; VNLL, ventral nucleus of lateral leminiscus; NLL, nucleus of lateral leminiscus; DC, dorsal cortex of IC; ICC, central nucleus of IC; LN, lateral nucleus of IC; IC, inferior colliculus (^*^P<0.05, ^**^P<0.0001).

GlyR IR in the IC was analyzed in three different regions namely, the DN, ICC and LN. According to the relative density, GlyR IR was markedly decreased in the IC of the E4 group compared with the SC group. GlyR IR was significantly (P<0.0001) decreased by 9.10% in the DC, from 104.01±2.71 in the SC group to 94.55±2.88 in the E4 group (Fig. 9D). GlyR IR in the ICC was also decreased significantly (P<0.0001) by 3.53%, from 101.18±1.71 in the SC group to 97.60±1.57 in the E4 group (Fig. 9D). Similarly, a 7.97% decrease was noted in the LN from 98.56±3.54 in the SC group to 90.70±3.75 in the E4 group, which was shown to be statistically significant (P<0.0001) (Fig. 9D).

#### v) GlyR immunoreactive cell count

**Figure 10:**
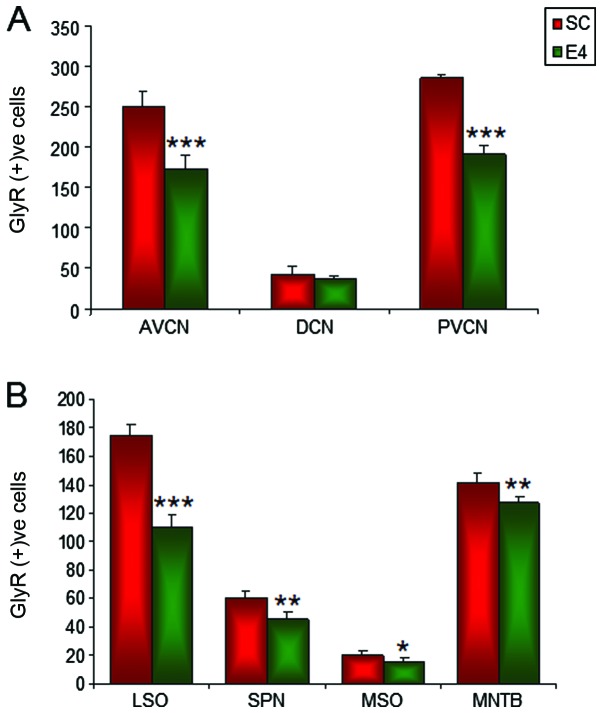
Graph demonstrating glycine receptor (GlyR) α_1_+α_2_ immunoreactive cell numbers in the CNC and SOC of sham control (SC) group (SAR 0 W/kg) and the group exposed to radiofrequency radiation (E4 group) (SAR 4.0W/kg) groups after three months of exposure to RF radiation at 835 MHz. The number of GlyR immunoreactive cells was significantly decreased in both the SOC and CN regions of the E4 group as compared with the SC group. The data shown are the means ± SD obtained from five different experiments. CNC, cochlear nuclear complex; AVCN, anteroventral cochlear nucleus; PVCN, posteroventral cochlear nucleus; DCN, dorsal cochlear nucleus; SOC, superior olivary complex; LSO, lateral superior olive; SPN, superior paraolivary nucleus; MSO, medial superior olive; MNTB, medial nucleus of the trapezoid body (^*^P<0.05, ^**^P<0.005, ^***^P<0.0001).

In the comparison of the GlyR immunoreactive cells in the SOC and CNC regions between the SC and E4 groups, the number of the GlyR immunoreactive cells in the E4 group was found to be significantly decreased. A 31.09% decrease (p<0.0001) in GlyR IR was observed in the AVCN from 250.2±18.29 in the SC group to 172.4±16.68 in the E4 group (Fig. 10A). Although a 14.08% decrease was observed in the DCN from 42.6±9.28 in the SC groiup to 36.6±4.27 in the E4 group, the difference was not statistically significant (Fig. 10A). The PVCN also displayed a significant difference (P<0.0001) with a 32.79% decrease in the number of GlyR immunoreactive cells from 285.4±3.36 in the SC group to 191.8±8.78 in the E4 group (Fig. 10A).

In the LSO region, there was a significant (P<0.0001) 36.85% decrease in GlyR IR from 174.2±7.49 in the SC group to 110±8.57 in the E4 group (Fig. 10B). A significant (P<0.005) 24.33% decrease in the number of GlyR immunoreactive cells was also observed in the SPN region, decreasing from 60±5.24 in the SC group to 45.48±4.72 in the E4 group (Fig. 10B). The MSO displayed a 23.23% decrease (P<0.05), from 19.8±2.86 in the SC group to 15.2±2.68 in the E4 group. The MNTB had the lowest decrease by far (10.15%; P<0.005), 141.8±6.18 in the SC group and 127.4±4.39 in the E4 group, although the IR decrease was more severe in the MNTB compared with the other SOC regions (Fig. 10B).

## Discussion

The structural and functional integrity of the neurons in the auditory brainstem nuclei is maintained by auditory input (37). Sensorineural differentiation and hair cell damage due to noise or chemical ototoxicity lead to changes in GlyR expression in the auditory brainstem nuclei (38–40).

The CNC is the major site through which binaural information converges in the CNS (19). Specific cell types within the cochlear nuclei receive morphologically distinct axosomatic endings of primary afferent fibers from the cochlea (21) and are highly vulnerable to pathological alterations from peripheral organs. The impairment of cochlear integrity brings about various morphological, biochemical and metabolic changes throughout the auditory system (41). Cochlear damage leads to a significant reduction in the number of Gly immunoreactive cells (42) and long-term deficiencies in glycinergic synaptic inhibition with the downregulation of post-synaptic GlyR activity in the VCN and LSO by unilateral cochlear ablation (38). All changes in the release, uptake and binding of Gly accompanying a significant shrinkage of the PVCN, AVCN and LSO on the ipsilateral side have been reported in the auditory brainstem associated with unilateral cochlear ablation (43). In accordance with the data presented in this study, prolonged monaural conductive hearing loss has been reported to lead to the downregulation of the GlyR α_1_ subunit (44). Furthermore, as previously demonstrated, by using *in situ* hybridization of the four GlyR subunits (α_1_, α_2_, α_3_ and β) in Fischer-344 rats, the mRNA expression of the α_1_ and β subunits in the AVCN decreased in the older age groups, which may contribute to central presbycusis (45).

The decrease in the number of GlyR immunoreactive neurons and puncta is also associated with hearing loss (33). In fact, a decrement in the number of GlyR immunoreactive cells and GlyR IR may be related to abnormalities in glycinergic inhibition by exposure to RF radiation as indicated by the present data. In the present study, a statistically significant decrease in GlyR IR in the cells, as well as in the cell number of the CNC (31.09% decrease in AVCN; 32.79% decrease in the PVCN) and the SOC (36.85% decrease in the LSO; 23.23% in the MSO) was also noted in the brainstems of the E4 group. Although no apoptosis was observed in the present study (data not shown), the ABR test demonstrated an elevation of the threshold, which was possibly the result of the induction of cochlear damage due to exposure to RF radiation.

A decrease in GlyR IR and the loss of GlyR immunoreactive neurons observed in the present study may result in cochlear damage by the downregulation of glycine release and post-synaptic GlyRs activity, which may weaken transmission at synapses made in the cochlear nucleus (CN) (43). Consecutively, the fibers from cells of the CN provide parallel ascending pathways to the SOC for processing different features of sound (19).

Improper functioning, particularly in the brainstem region, in the SOC nuclei has been reported to decrease glycine levels, which may lead to hearing impairment (22). SOC as a convergence site of the binaural input is likely to be involved in sound localization (46). In the SOC of the E4 group, a significant decrease in the number of GlyR immunoreactive cells and puncta on the somas of the principal cells, which could be the possible source of inhibitory input of SOC, may contribute to altered receptor activity and auditory functions, which is often an adjunct to hearing loss (43). Given the role of SOC in the processing of interaural phase disparity (47) and interaural time differences (28), a disturbance in the GlyR expression in the auditory brainstem may be related to hearing deficits and auditory dysfunction in the E4 group in this study. In addition, hearing impairment may also disrupt the afferent projections from the DNLL to the ICC (48,49). According to our data, the decrease in GlyR IR in the soma of DNLL was also statistically significant (p<0.0001).

GlyR expression, which plays a major role in mediating the inhibitory input of hearing processes, is considerably decreased under the experimental conditions that were used in this study (the conditions the mice were subjected to). The decrease in GlyR IR and the number of GlyR immunoreactive cells in the auditory brainstem, as observed in the E4 group, certainly points towards the vulnerability of the auditory brainstem region to exposure to RF radiation. Hence, prolonged exposure to RF radiation could affect the auditory brainstem circuit, leading to hearing impairment by the decrease of an inhibitory source, such as Gly through intriguing signaling cascades.

The scientific investigation of the possible health consequences of RF radiation has become an issue of international interest and public debate, since all populations are exposed to varying degrees of RF radiation. However, the identification of the potential health effects of exposure to RF radiation is a difficult task due to rapid advances in the application of RF fields in our environment, about which anxiety and assumption are spreading. However, according to the present study, the controversial association between exposure to RF radiation and disease has been addressed, although, to date, no adverse health effects have been established as being caused by the use of commercial mobile phones. Further research is required in order to identify the potential effects of RF radiation and the exact molecular signal cascades involved and obtain a better assessment of the pathology of hearing impairment.

## References

[1] Frey AH (1998). Headaches from cellular telephones: are they real and what are the implications. Environ Health Perspect.

[2] Borbély AA, Huber R, Graf T, Fuchs B, Gallmann E, Achermann P (1999). Pulsed high-frequency electromagnetic field affects human sleep and sleep electroencephalogram. Neurosci Lett.

[3] Manikonda PK, Rajendra P, Devendranath D, Gunasekaran B, Channakeshava, Aradhya RS, Sashidhar RB, Subramanyam C (2007). Influence of extremely low frequency magnetic fields on Ca^2+^signaling and NMDA receptor functions in rat hippocampus. Neurosci Lett.

[4] Mausset AL, de Seze R, Montpeyroux F, Privat A (2001). Effects of radiofrequency exposure on the GABAergic system in the rat cerebellum: clues from semiquantitative immunohistochemistry. Brain Res.

[5] Salford LG, Brun AE, Eberhardt JL, Malmgren L, Persson BR (2003). Nerve cell damage in mammalian brain after exposure to microwaves from GSM mobile phones. Environ Health Perspect.

[6] Maskey D, Pradhan J, Aryal B, Lee CM, Choi IY, Park KS, Kim SB, Kim HG, Kim MJ (2010). Chronic 835-MHz radiofrequency exposure to mice hippocampus alters the distribution of calbindin and GFAP immunoreactivity. Brain Res.

[7] Bas O, Odaci E, Kaplan S, Acer N, Ucok K, Colakoglu S (2009). 900 MHz electromagnetic field exposure affects qualitative and quantitative features of hippocampal pyramidal cells in the adult female rat. Brain Res.

[8] Ammari M, Brillaud E, Gamez C, Lecomte A, Sakly M, Abdelmelek H, de Seze R (2008). Effect of a chronic GSM 900 MHz exposure on glia in the rat brain. Biomed Pharmacother.

[9] Maskey D, Kim HJ, Kim HG, Kim MJ (2012). Calcium-binding proteins and GFAP immunoreactivity alterations in murine hippocampus after 1 month of exposure to 835 MHz radiofrequency at SAR values of 1.6 and 4.0 W/kg. Neurosci Lett.

[10] Mausset-Bonnefont AL, Hirbec H, Bonnefont X, Privat A, Vignon J, de Seze R (2001). Acute exposure to GSM 900-MHz electromagnetic fields induces glial reactivity and biochemical modifications in the rat brain. Neurobiol Dis.

[11] Vater M, Habbicht H, Kössl M, Grothe B (1992). The functional role of GABA and glycine in monaural and binaural processing in the inferior colliculus of horseshoe bats. J Comp Physiol A.

[12] Caird DM, Palmer AR, Rees A (1991). Binaural masking level difference effects in single units of the guinea pig inferior colliculus. Hear Res.

[13] Wu SH, Kelly JB (1994). Physiological evidence for ipsilateral inhibition in the lateral superior olive: synaptic responses in mouse brain slice. Hear Res.

[14] Kotak VC, Sanes DH (1996). Developmental influence of glycinergic transmission: regulation of NMDAreceptor-me diated EPSPs. J Neurosci.

[15] Sanes DH, Takács C (1993). Activity-dependent refinement of inhibitory connections. Eur J Neurosci.

[16] Wenthold RJ, Huie D, Altschuler RA, Reeks KA (1987). Glycine immunoreactivity localized in the cochlear nucleus and superior olivary complex. Neuroscience.

[17] Moore MJ, Caspary DM (1983). Strychnine blocks binaural inhibition in lateral superior olivary neurons. J Neurosci.

[18] Wenthold RJ, Altschuler RA, Bobbin RP, Clopton BM, Hoffman DW (1991). Neurotransmitters of brainstem auditory nuclei. Neurobiology of Hearing: The Central Auditory System.

[19] Cant NB, Benson CG (2003). Parallel auditory pathways: projection patterns of the different neuronal populations in the dorsal and ventral cochlear nuclei. Brain Res Bull.

[20] Webster DB, Trune DR (1982). Cochlear nuclear complex of mice. Am J Anat.

[21] Ryugo DK, Parks TN (2003). Primary innervation of the avian and mammalian cochlear nucleus. Brain Res Bull.

[22] Buras ED, Holt AG, Griffith RD, Asako M, Altschuler RA (2006). Changes in glycine immunoreactivity in the rat superior olivary complex following deafness. J Comp Neurol.

[23] Kavanagh GL, Kelly JB (1992). Midline and lateral field sound localization in the ferret (*Mustela putorius*): contribution of the superior olivary complex. J Neurophysiol.

[24] O’Neill WE, Zettel ML, Whittemore KR, Frisina RD (1997). Calbindin D-28k immunoreactivity in the medial nucleus of the trapezoid body declines with age in C57BL/6, but not CBA/CaJ, mice. Hear Res.

[25] Irvine DRF, Popper AN, Fay RR (1992). Physiology of the auditory brainstem. The Mammalian Auditory Pathway: Neurophysiology.

[26] Oliver DL, Huerta MF, Webster DB, Popper AN, Fay RR (1992). Inferior and superior colliculi. The Mammalian Auditory Pathway: Neuroanatomy.

[27] Shneiderman A, Henkel CK (1987). Banding of lateral superior olivary nucleus afferents in the inferior colliculus: a possible substrate for sensory integration. J Comp Neurol.

[28] Grothe B, Sanes DH (1993). Bilateral inhibition by glycinergic afferents in the medial superior olive. J Neurophysiol.

[29] Wu SH, Kelly JB (1995). Inhibition in the superior olivary complex: pharmacological evidence from mouse brain slice. J Neurophysiol.

[30] Savtchouk I, Liu SJ (2011). Remodeling of synaptic AMPA receptor subtype alters the probability and pattern of action potential firing. J Neurosci.

[31] Banks MI, Smith PH (1992). Intracellular recordings from neurobiotin-labeled cells in brain slices of the rat medial nucleus of the trapezoid body. J Neurosci.

[32] Kulesza RJ, Berrebi AS (2000). The superior paraolivary nucleus of the rat is a GABAergic nucleus. J Assoc Res Otolaryngol.

[33] Willott JF, Turner JG (2000). Neural plasticity in the mouse inferior colliculus: relationship to hearing loss, augmented acoustic stimulation, and prepulse inhibition. Hear Res.

[34] Ozturan O, Erdem T, Miman MC, Kalcioglu MT, Oncel S (2002). Effects of the electromagnetic field of mobile telephones on hearing. Acta Otolaryngol.

[35] Kizilay A, Ozturan O, Erdem T, Kalcioglu T, Miman MC (2003). Effects of chronic exposure of electromagnetic fields from mobile phones on hearing in rats. Auris Nasus Larynx.

[36] Aran JM, Carrere N, Dulou PE, Larrieu S, Letenneur L, Veyret B, Dulon D (2004). Effects of exposure of the ear to GSM microwaves: in vivo and in vitro experimental studies. Int J Audiol.

[37] Rubel EW, Hyson RL, Durham D (1990). Afferent regulation of neurons in the brain stem auditory system. J Neurobiol.

[38] Potashner SJ, Suneja SK, Benson CG (2000). Altered glycinergic synaptic activities in guinea pig brain stem auditory nuclei after unilateral cochlear ablation. Hear Res.

[39] Vale C, Sanes DH (2002). The effect of bilateral deafness on excitatory and inhibitory synaptic strength in the inferior colliculus. Eur J Neurosci.

[40] Whiting B, Moiseff A, Rubio ME (2009). Cochlear nucleus neurons redistribute synaptic AMPA and glycine receptors in response to monaural conductive hearing loss. Neuroscience.

[41] Syka J (2002). Plastic changes in the central auditory system after hearing loss, restoration of function, and during learning. Physiol Rev.

[42] Potashner SJ, Suneja SK, Benson CG (1997). Regulation of D-aspartate release and uptake in adult brain stem auditory nuclei after unilateral middle ear ossicle removal and cochlear ablation. Exptl Neurol.

[43] Suneja SK, Benson CG, Potashner SJ (1998). Glycine receptors in adult guinea pig brain stem auditory nuclei: regulation after unilateral cochlear ablation. Exp Neurol.

[44] Wang H, Yin G, Rogers K, Miralles C, De Blas AL, Rubio ME (2011). Monaural conductive hearing loss alters the expression of the GluA3 AMPA and glycine receptor α1 subunits in bushy and fusiform cells of the cochlear nucleus. Neuroscience.

[45] Krenning J, Hughes LF, Caspary DM, Helfert RH (1998). Age-related glycine receptor subunit changes in the cochlear nucleus of Fischer-344 rats. Laryngoscope.

[46] Masterton B, Jane JA, Diamond IT (1967). Role of brainstem auditory structures in sound localization. I. Trapezoid body, superior olive, and lateral lemniscus. J Neurophysiol.

[47] Spitzer MW, Semple MN (1995). Neurons sensitive to interaural phase disparity in gerbil superior olive: diverse monaural and temporal response properties. J Neurophysiol.

[48] Franklin SR, Brunso-Bechtold JK, Henkel CK (2006). Unilateral cochlear ablation before hearing onset disrupts the maintenance of dorsal nucleus of the lateral lemniscus projection patterns in the rat inferior colliculus. Neuroscience.

[49] Franklin SR, Brunso-Bechtold JK, Henkel CK (2008). Bilateral cochlear ablation in postnatal rat disrupts development of banded pattern of projections from the dorsal nucleus of the lateral lemniscus to the inferior colliculus. Neuroscience.

